# A Germline Mutation in ATR Is Associated With Lung Adenocarcinoma in Asian Patients

**DOI:** 10.3389/fonc.2022.855305

**Published:** 2022-05-31

**Authors:** Guangyao Bao, Xiaojiao Guan, Jie Liang, Yao Yao, Yifan Xiang, Tian Li, Xinwen Zhong

**Affiliations:** ^1^ Department of Thoracic Surgery, First Affiliated Hospital, China Medical University, Shenyang, China; ^2^ Department of Pathology, Shengjing Hospital, China Medical University, Shenyang, China; ^3^ School of Basic Medicine, Fourth Military Medical University, Xi’an, China

**Keywords:** familiar lung cancer, ATR, sequencing, germline mutation, lung adenocarcinoma

## Abstract

**Background:**

Familial lung cancer (FLC) accounts for 8% of lung adenocarcinoma. It is known that a few germline mutations are associated with risk increasing and may provide new screening and treatment option. The goal of this study is to identify an FLC gene among three members of an FLC family.

**Methods:**

To uncover somatic and embryonic mutations linked with familial lung cancer, whole exome sequencing was done on surgical tissues and peripheral blood from three sisters in a family diagnosed with pulmonary lung adenocarcinoma (LUAD). At the same time, single-cell RNA sequencing (scRNA-seq) and bulk RNA sequencing data in public databases were enrolled to identify specific gene expression level.

**Results:**

Ataxia Telangiectasia and Rad3-Related Protein (ATR) gene C.7667C >G (p.T2556S) mutation were found in 3 patients with familial lung cancer. Whole-genome sequencing revealed that the three sisters exhibited similar somatic mutation patterns. Besides ATR mutations, common mutated genes (BRCA1, EGFR, and ROS1) that characterize LUAD were also found in 5 tumor samples. Analysis for the ATR expression in LUAD patients by single-cell sequencing data, we found ATR expression of tumor patients at high level in immune cells when compared with normal patients, but the expression of ATR in stromal cells has the opposite result.

**Conclusion:**

We found a germline mutation in the ATR gene in three sisters of a Chinese family affected by familial lung cancer, which may be a genetic factor for lung cancer susceptibility.

## Introduction

Lung adenocarcinoma is the primary cause of cancer-related death worldwide, with an estimated 1.8 million fatalities each year (1). Researchers are still considering possible links involving lung cancer risk. Although smoking is still the leading cause of lung cancer, accounting for 80 to 90 percent of all cases, genetic susceptibility may play a role in lung cancer in some situations (2). Previous studies have demonstrated that genetic susceptibility was thought to be closely related to the development of familial lung cancer (3, 4). However, the risk factors for hereditary lung cancer are unknown at this time. Several research has looked into the function of germline mutations, primarily selected somatic mutations, in lung cancer susceptibility (5–7).

Ataxia Telangiectasia and Rad3 related (ATR) kinase plays a crucial role in the repair of replication-associated DNA damage (8). The study of cell cycle checkpoint signaling through ATR, as well as the related pathways implicated in oncogenesis and cancer progression, has resulted in the discovery and development of effective and selective ATR inhibitors (ATRi) (9). According to recent research of inherited germline mutations in a Chinese population with lung cancer assessed using an NGS, the majority of the discovered germline mutations (85.5 percent) were implicated in DNA damage repair (DDR) pathways (10). There have previously been reports of rare events in lung cancer patients, which may be related to the genetic susceptibility to lung cancer (11). As a high-throughput method, next-generation sequencing (NGS) has become routine in clinical practice and has altered the clinical management of lung cancer (12, 13). This method enables for massively parallel characterization of thousands of cells at the transcriptome level. In this study, we identified an interesting ATR mutation by whole-exome sequencing in three sisters of this family with lung cancers.

## Material and Methods

### Patients Data

**Figure 1:**
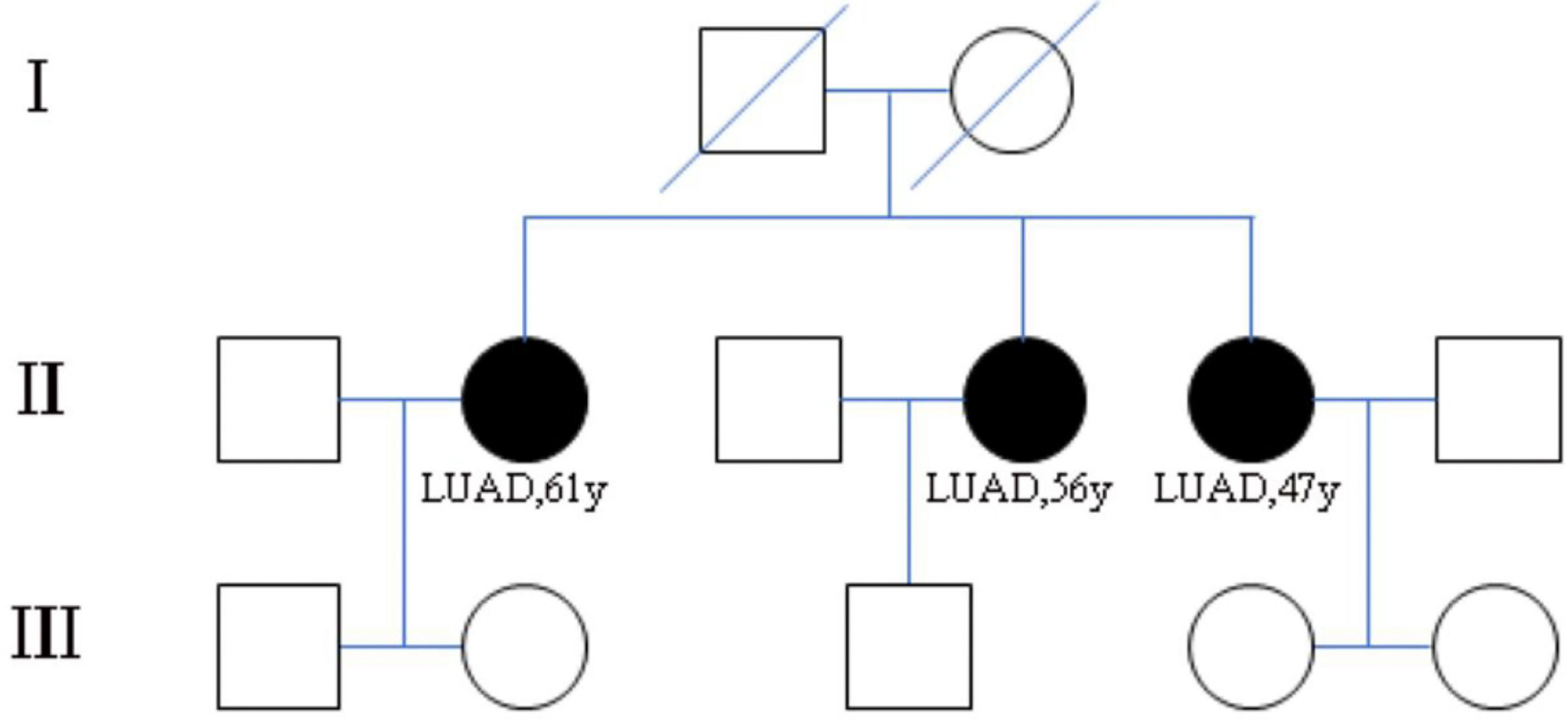
Pedigree of family (case 3) with multiple cases of lung adenocarcinoma. II-2, II-4, II-5 are sisters and have been diagnosed as lung adenocarcinoma. I-1 and I-2 are their parents and have passed away. The other members of this family have no history of lung tumor.

**Figure 2:**
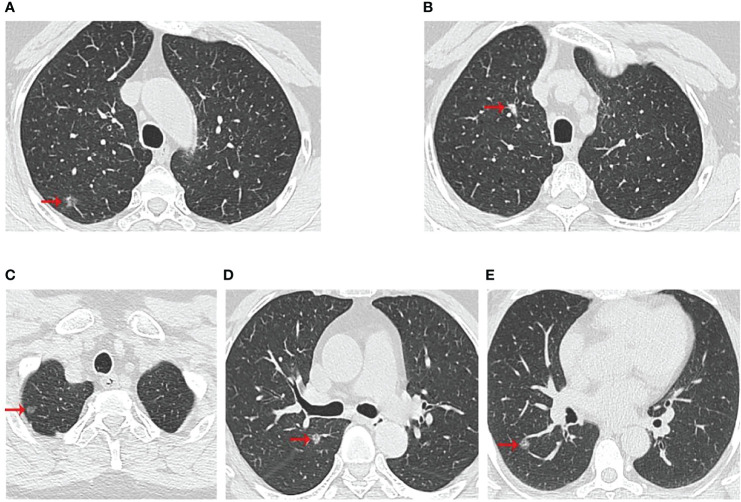
Computed tomographic scan of chest at diagnosis of three patients. **(A, B)** were II-2 CT scan results, which showed two nodules in the upper right lobe; **(C, D)** were II-4 CT scan results, which showed two nodules in the upper lobe of the right lung; **(E)** was II-5 CT scan results with a right lower lobe nodule. The red arrows indicated the location of the lesions.

The three patients were sisters and were treated successively in the Thoracic Surgery Department of the First Hospital of China Medical University due to lung nodules. We have drawn the pedigree of the lung adenocarcinoma family (**Figure 1**). The first woman was a 61-year-old female with 40 years of smoking history (II -2), who was diagnosed with micro-invasive adenocarcinoma and invasive adenocarcinoma of two nodules in the upper right lobe (**Figures 2A, B**). Another 56-year-old female, non-smoker, with 2 nodules in the upper right lobe (II -4), was pathologically confirmed to be adenocarcinoma *in situ* and invasive adenocarcinoma after surgery (**Figures 2C, D**). Invasive adenocarcinoma of the right lower lobe (II-5) was diagnosed in a 47-year-old non-smoker sister of the family (**Figure 2E**). The other members of this family have no history of lung tumor. This study was approved by the ethics committees of the First Hospital of China Medical University. The patient signed written informed consent, and we kept the patient’s identity protection for the duration of the study.

### Whole-Exome Sequencing (WES)

In total, peripheral blood, cancer, para-cancerous samples, and clinical data of three patients were collected for whole-exome sequencing (WES) in this study. NEBNext dsDNA Fragmentase (NEB, Ipswich, MA, USA) was used to extract and fragment genomic DNA from blood and cancer or para-cancer (normal tissues adjacent to cancer) tissues, followed by DNA end mending. After being detailed, end-repaired DNA segments were ligated with the NEBNext adaptor (NEB, Ipswich, MA, USA). Biotinylated RNA library baits and magnetic beads were coupled with the barcoded library to detect particular areas using the SureSelect Human All Exon V6 Kit (Agilent Technologies, Palo Alto, Calif.). On an Illumina X-ten system, the acquired sequences were amplified further for 150bp paired-end sequencing (Illumina, San Diego, CA, USA). Readings of high quality that passed the Illumina filter were kept for further processing.

### Mutation Analysis

Using fastp, raw reads would be processed to make high-quality clean reads (14). To match the clean reads from each sample to the reference genome, the Burrows-Wheeler Aligner (BWA) (15) was employed (GRCh38.p12). The Genome Analysis Toolkit (GATK) (16) was used to call variants for multi-sample analyses. For multi-sample variant calling, local realignment, and base quality score recalibration, Unified Genotyper was employed. To assess the frequency of each SNP, the software package ANNOVAR (17) was used to align and annotate SNPs or InDels.Variants related to diseases were annotated with Clinvar database (http://www.clinvar.com/).

### Bioinformatic Analysis

Gene expression profiles of 483 tumor samples and 347 normal samples in LUAD were conducted by GEPIA database (18). RNA-seq data and its relevant clinical information and mutation data of 561 LUAD samples were received from The Cancer Genome Atlas (TCGA) database (https://portal.gdc.cancer.gov/). Survival analysis was conducted by “survival” R package. The “maftools” R package was used to find high-frequency mutation genes. The GEO database (http://www.ncbi.nlm.nih.gov/geo/) was used to download raw UMI counts per gene, as well as the sample and cluster annotations of 11 human LUAD and 11 human normal lung samples from Kim et al’s research (19). The raw counts were normalized using the “scran” R program, and then cluster annotation was performed using the “SingleR” R tool.

## Results

### Molecular Characterization of Genetic ATR Mutations

We performed whole-exome sequencing on surgical samples and peripheral blood to discover variants associated with familial lung cancer. Then, we applied the following filters for the variants called by whole exome sequencing to identify the most significant germline mutations: (1) all affected family members must have mutations; (2) variations in the SNP databases with a minor allele frequency (MAF) greater than 0.5 percent were eliminated from further research, cause they are more likely to be functional neutral polymorphisms (the SNP databases that we used for filtering are Clinvar from Complete Genomics); (3) the variations must be missense or nonsense mutations or indels that cause in alterations or a shortened protein.; (4) bioinformatics algorithms such as PolyPhen-2 (20), SIFT (21), or MutationTaster must forecast that these mutations are functionally “non-benign” or “intolerable” and are linked to cancer (22). We summarized and annotated the mutation data of three patients (**Table 1**), and seven candidate variants were discovered in three patients of this family, only the ATR c.7667C>G (p.T2556S) mutation is the most important germline mutation in this family.

**Table 1:** Characteristics of the seven candidate variants for familial lung cancer.

Gene	Genomic Position	Genomic Mutation	dbSNP	Protein Alteration	Function	ClinVar Assessment
ATR	Chr3: 142453222	c.7667C>G	rs200490116	p.Thr2556Ser	Missense	Uncertain significance
SMG5	Chr1: 156263493	c.1933A>G	rs200093957	p.Lys645Glu	Missense	Not listed
RAB3GAP2	Chr1: 220164744	c.3143A>G	rs151244742	p.His1048Arg	Missense	Likely benign
EI24	Chr11: 125575342	c.122G>A	rs559933286	p.Arg41His	Missense	Not listed
XDH	Chr2: 31350061	:c.2794G>A	rs141291583	p.Ala932Thr	Missense	Not listed
PTPRA	Chr20: 3035681	c.2017G>A	rs61742029	p.Val673Ile	Missense	Not listed
GSTZ1	Chr14:77326864	c.94G>A	rs7975	p.Glu32Lys	Missense	Stop Gained

* indicates nucleotide number and translation termination (stop) codon.

### Analysis Somatic Mutations in FLC

**Figure 3:**
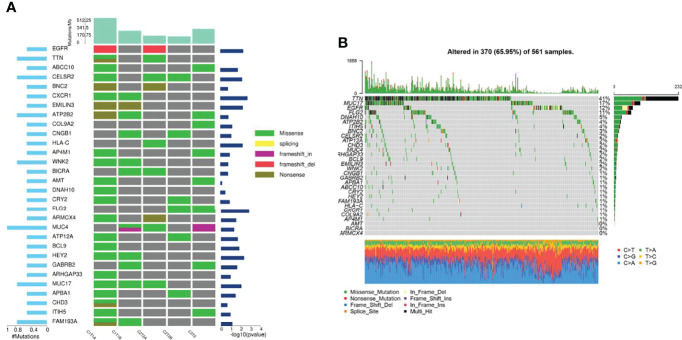
Gene mutation characteristics of three patients and in TCGA database. **(A)** High-frequency mutation genes of three lung adenocarcinoma patients; **(B)** Mutation spectrum of high-frequency mutation genes in TCGA mutation data.

Then, the results of somatic mutations in five tumor lesions of three patients were thoroughly studied. The somatic SNV/INDEL mutation spectrum and CNV mutation spectrum of the three lung cancer patients in this investigation were mapped (**Supplementary Figure S1**). Moreover, we searched the reported driver gene data to screen out the known driver genes in the tumor sample based on Cancer Gene Census (http://cancer.sanger.ac.uk/census), MDG125 (23), SMG127 (24), CDG291 (25) database and we noticed that somatic mutations frequently found in this family, such as EGFR, BRCA1 and ROS1 were also observed (**Table 2**). Furthermore, new candidate driver genes of lung adenocarcinoma, like PNCK and IP6K2 could also be found in this family (**Table 3**). Finally, we used MuSiC software (26) to find genes with higher mutation frequencies of the three patients, we found the top thirty high-frequency mutation genes (**Figure 3A**) and we summarized the mutation information of these genes in the TCGA mutation data, and we found that AMT, BICRA, ARMCX4 are high-frequency mutation genes unique to these three patients (**Figure 3B**).

**Table 2:** Known somatic mutation patterns in three patients.

Gene	Chr	Start	End	Variation Type	Ref	Mut	Patients
BRCA1	17	43104073	43104089	Deletion	AAAAAAAAGAAAAGAAG	–	II-4 Tumor B
EGFR	7	55173052	55173052	SNP	G	T	II-2 Tumor A
EGFR	7	55202802	55202802	SNP	G	A	II-2 Tumor B
EGFR	7	55174774	55174788	Deletion	AATTAAGAGAAGCAA	–	II-4 Tumor A
EGFR	7	55174773	55174787	Deletion	GAATTAAGAGAAGCA	–	II-4 Tumor A
ROS1	6	117356812	117356812	SNP	G	T	II-2 Tumor A
ROS1	6	117341369	117341369	SNP	C	A	II-5

**Table 3:** Predict Driver Genes in Three FLC Patients.

PNCK	IP6K2	CLEC18C	PARD3	TTN	CMTM3	TMPRSS6
PCBP4	CNOT10	RAB43	DZANK1	C1orf43	KIF9	INPP5B
SUOX	ASIC3	PLCD4	NPAS3	SPTBN2	CDC25B	ARHGAP33
SLC6A2	LTF	SLC26A11	DNAH10	MUC17	AP4M1	GLG1
GSN	AMT	CHRNA2	RUBCNL	KCNH6	ANK2	CYTH2
NAV2	ADORA1	MYO7A	ARHGAP25	LTBP3	MASP1	PRSS54
AURKB	KLHDC2	WIPF1	FBLN7	TRAIP	PHF12	MVK
CRY2	ITGA7	CLCN2	ARHGEF3	KLHL13	EHMT1	PTK7
SPHK2	SLC4A11	SLFN13	CCDC120	TIMMDC1	LOXL3	SYNGAP1
HLA-G	ARHGAP27	SYNE2	SORL1	RPH3A	EYA2	RAB5C
LIMK1	EPN2	SEC16A	FARSA	TMEM267	COL6A3	IPO5
ZSWIM8	CNGB1	GRIN1	CAPN1	WSCD1	PCGF2	TM7SF2
LMCD1	RBBP7	SEMA4A	PTPRA	KDM4C	ZDHHC8	FDXR
ZAN	RASA4	PSMD13	HOMER3	TPI1	HLA-C	SKIL
ATP2B2	PRPF31	DLGAP4	SLC8B1	TPM2	TADA2B	STARD3NL
ABCC12	TRIM2	RIC8B	GABRB2	CXCR2	GRB10	TBC1D16
MAP3K19	TAF1C	SORBS1	DTNB	CACNA1A	POLDIP3	CES2
OBSCN	EPS8L2	SYVN1	BSDC1	RNF32	STK25	KCNT1
ATXN2L	TLE2	MVB12A				

### ATR Expression in LUAD Specimen

**Figure 4:**
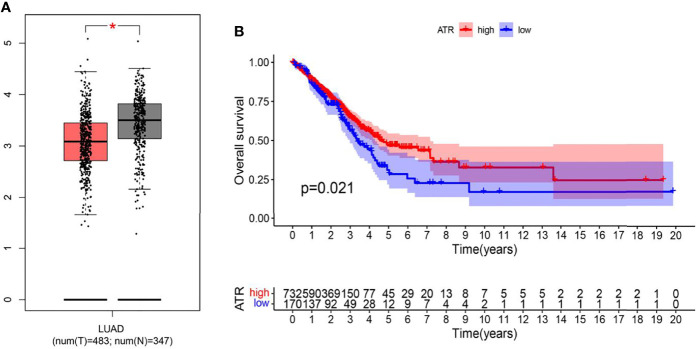
The expression and prognostic differences of ATR in TCGA database. **(A)** The expression of ATR in LUAD and normal tissues in GEPIA database (Tumor: 483; Normal: 347) (* indicates p < 0.05); **(B)** Down regulation of ATR is associated with harmful outcome in TCGA database (Tumor: 535; Normal: 59) (p = 0.021).

**Figure 5:**
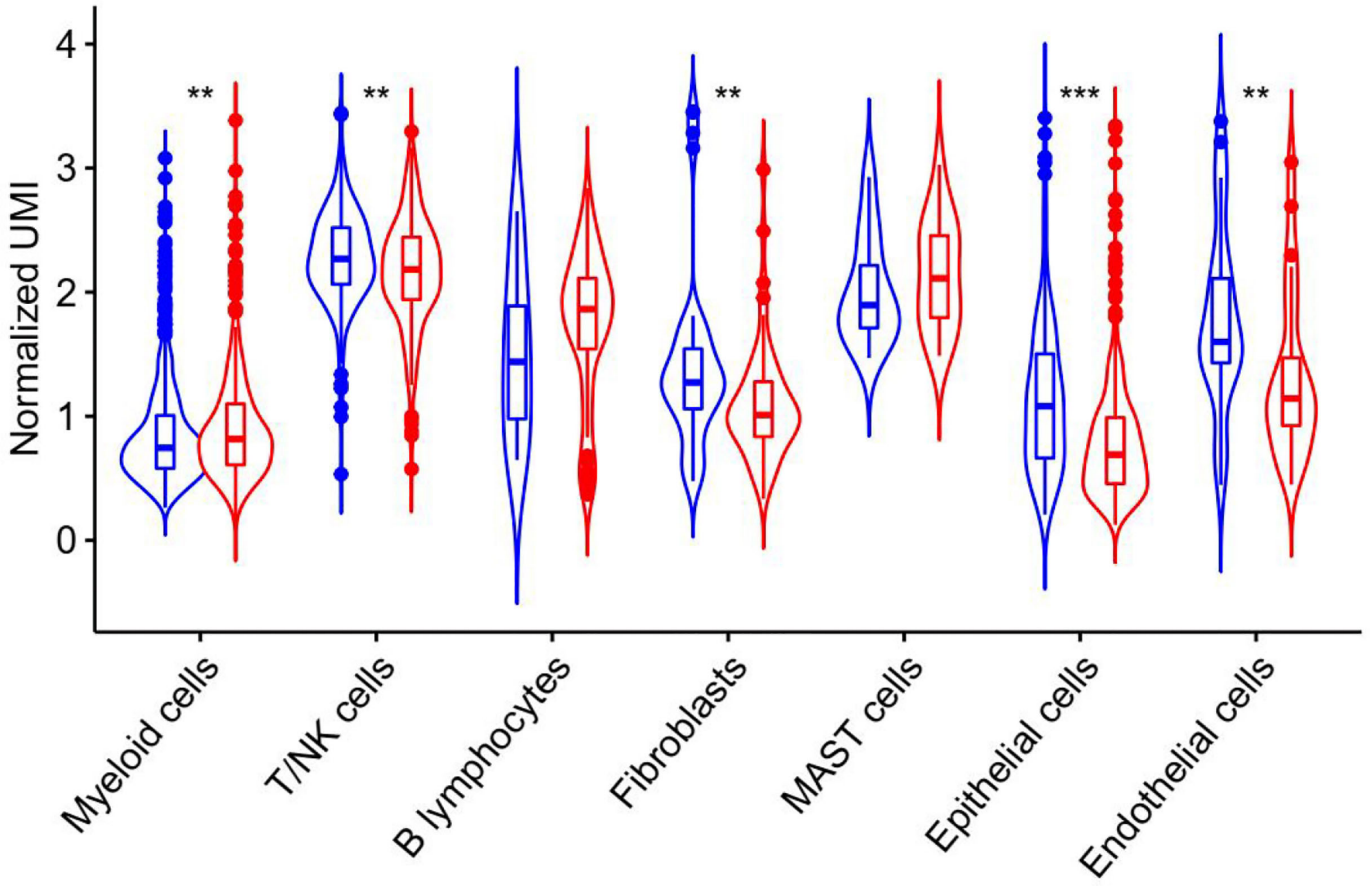
ATR expression in unique molecular identifiers in the indicated cell types from single-cell RNA-Seq data derived from human LUAD specimen. Blue represents the expression of ATR in each component of normal tissue, and red represents the expression of ATR in each component of tumor tissue.

ATR is required for maintaining genome integrity during DNA replication and plays a key role in avoiding replication stress at toxic levels, according to previous research. Because of these critical functions, cancers infrequently involve loss-of-function mutations in the ATR pathway, and a subset of tumors with particular mutations is more vulnerable to ATR pathway inhibition than normal cells (27). The underlying mechanism of ATR expression in lung adenocarcinoma requires further study, hence we analyzed ATR related expression and prognosis in cases of lung adenocarcinoma. As demonstrated in **Figure 4**, the expression of ATR was downregulated in LUAD patients as compared to normal individuals, and the downregulation of ATR expression was related to poor prognosis. Moreover, we used single-cell sequencing data to further explore the expression of ATR in designated cell types of lung adenocarcinoma and found the expression of ATR in stromal cells (Fibroblasts, Epithelial cells, Endothelial cells) of tumor patients is at a lower level when compared with normal patients, but the expression of ATR in the immune cells (Myeloid cells, B lymphocytes, MAST cells) of tumor patients is at a high level, except for T/NK cells (**Figure 5**).

### Analysis of Common Germline Genes in Chinese LUAD Patients

Next, we analyzed the whole-exome sequencing data of 2706 patients to uncover the germline gene mutations in the Chinese LUAD cohort. We then screened out 27 germline gene mutations and listed their mutation frequencies and mutation sites (**Table 4**). Among the relatively common mutant genes, including MLH1 (1.03474%), BRCA (0.77605%), STK11 (0.62823%), CHEK2 (0.44346%), TP53 (0.44346%), have been reported related to lung cancer. Meanwhile, multiple mutation sites of these germline gene mutations were also revealed. For example, we have detected 3 different mutation sites in the germline mutation of MLH1, including c.649C>T (p.R217C), p.Q701K (c. C2101A), and p.V384D (c.T1151A).

**Table 4:** Common germline gene mutations of 2706 Chinese LUAD patients.

Germline Mutation Gene	Mutation Frequency	Mutation Sites
MLH1	1.03474%	c.649C>T(p.R217C), p.Q701K (c.C2101A), p.V384D (c.T1151A)
BRCA2	0.77605%	c.2830A>T(p.K944*), c.671-1G>A, c.956dupA(p.N319Kfs*8)
STK11	0.62823%	p.F354L (c.C1062G)
CHEK2	0.44346%	p.H371Y (c.C1111T), c.1245dup(p.K416Qfs*22)
TP53	0.44346%	c.91G>A(p.V31I), p.V31I (c.G91A), c.7516-1G>A
ATM	0.29564%	c.3602_3603delTT(p.F1201Wfs*3), p.S1042R (c.A3124C)
BRCA1	0.25868%	c.671-1G>A
CDH1	0.25868%	p.T340A (c.A1018G)
MSH6	0.25868%	c.1406A>G(p.Y469C)
MUTYH	0.18477%	c.55C>T(p.R19*)
PALB2	0.18477%	c.2480_2481del(p.T827Mfs*6)
PMS2	0.18477%	c.1A>G(p.M1)?
RAD51D	0.18477%	c.270_271dup(p.K91Ifs*13)
ATR	0.11086%	c.4681C>T(p.Q1561*)
BRIP1	0.11086%	c.918+1G>A
MSH2	0.11086%	p.L390F (c.C1168T)
NBN	0.11086%	c.1651dupA(p.R551Kfs*5)
EPCAM	0.07391%	c.556-14A>G
FANCA	0.07391%	c.1900+1G>T
POLE	0.07391%	c.G4952+1T
RET	0.07391%	p.V804M (c.G2410A)
SDHA	0.07391%	c.1A>T(p.M1)?
VHL	0.07391%	p.W8X (c.G23A)
APC	0.03695%	c.3374T>C(p.V1125A)
FANCI	0.03695%	c.504-1G>A
MEN1	0.03695%	c.1A>G(p.M1)?
SDHB	0.03695%	c.200+1G>C

* indicates nucleotide number and translation termination (stop) codon.

## Discussion

Carcinogenesis is a multi-factor and multi-stage process and gene mutation is an important cause of cancer occurrence and development (28). Germline mutations including somatic mutation and germline mutations. Germline mutations which occur in the reproductive cells, it can be passed on to the next generation, affecting every cell in the next generation. However, most studies on tumor mutations have focused on somatic mutations. The mining and identification of tumor germline mutations are very limited, with only a few cases reported in lung adenocarcinoma. As a result, the identification of novel germline mutations is critical for both fundamental research and clinical cancer treatment (29).

In previous studies, most of the genes with germline mutations in lung adenocarcinoma were typical oncogenes and proto-oncogenes, which included BRCA2, CHECK2, CDKN2, BAP1, EGFR (30–32). The genomic alternations of these genes were thought to be important in familial lung cancer. Then, with the development of high-throughput sequencing data, germline altered variants in other genes (MAST1, CENPE, LCT) (33–35) were identified to exhibit an essential role in the development of lung adenocarcinoma, and they were suspected to be related to the development of lung cancer through whole-exome or genome sequencing of samples from many lung cancer patients.

In this study, we continue to focus on the germline mutation of genes in lung adenocarcinoma and we have identified seven different variants (SMG5, RAB3GAF2, EI24, XDH, PTPRA, ATR, GSTZ1) in three sibling patients suffering from lung cancer. The most significant is a rare somatic mutation in the ATR gene named c.7667C>G (p.T2556S), and this ATR gene variation has never been observed in genome and exome research within combined populations. Then, we analyzed the expression and prognosis of ATR in lung adenocarcinoma. Compared with normal patients, the expression of ATR was down-regulated in LUAD, and the down-regulated expression of ATR was associated with poor prognosis. Furthermore, we employed single-cell sequencing data to investigate the expression of ATR in lung adenocarcinoma cell types. Compared with normal patients, we found that the expression level of ATR in the stromal cells (fibroblasts, epithelial cells, and endothelial cells) of tumor patients was low. In addition to T/NK cells, the expression level of ATR in immune cells (myeloid cells, B lymphocytes, MAST cells) of tumor patients is higher. In view of this, we have reason to believe that germline ATR c.7667C>G (p.T2556S) mutation may lead to familial clusters of lung adenocarcinoma and differentially expressed ATR may play an essential role in the occurrence and development of lung adenocarcinoma. Moreover, based on somatic mutation data, we found common driver gene mutations such as EGFR, BRCA1, ROS1, etc. We predicted 136 new driver gene mutations such as PNCK, IP6K2, CLEC18C, etc. through OncodriveCLUST software (36). These gene mutations may be secondary somatic mutations based on germline ATR c.7667C>G (p.T2556S) mutations, which eventually lead to the occurrence and progression of lung adenocarcinoma.

ATR, as a necessary gene in the DNA damage repair pathway, is involved in the coordination of cell-cycle transitions, DNA replication, DNA repair, and apoptosis (37). Previous studies have shown that germline gene mutations that occur in the DNA damage repair pathway often irreversibly lead to tumors (38, 39). Furthermore, germline abnormalities in DNA repair genes lead to advanced solid tumor (such as prostate, ovarian, pancreatic, and breast cancer) susceptibility to poly ADP ribose polymerase (PARP) inhibitors (40–42). To our knowledge, this is the first such case report confirming the presence of c.7667C>G (p.T2556S), a germline ATR mutation, in a family with three occurrences of lung adenocarcinoma The resulting amino acid mutation, p.Thr2556Ser, was detected in the ATR’s conserved Phosphoinositide 3- and 4-kinase domain, which is involved in biological processes such as cell growth, proliferation, differentiation, motility, survival, and intracellular trafficking (43).

The population frequency in the entire population is 0.0399361 percent, suggesting that this ATR germline mutation may be a genetic susceptibility factor for lung adenocarcinoma. Our findings contribute to the understanding of the genesis and mechanism of lung cancer and may give clues for oncology treatment strategies and cancer prevention.

## Data Availability Statement

The datasets presented in this study can be found in online repositories. The names of the repository/repositories and accession number(s) can be found below: NCBI [accession: PRJNA800743].

## Ethics Statement

The studies involving human participants were reviewed and approved by ethics committees of the First Hospital of China Medical University. The patients/participants provided their written informed consent to participate in this study. Written informed consent was obtained from the individual(s) for the publication of any potentially identifiable images or data included in this article.

## Author Contributions

GB conceived and designed the study, and drafted the manuscript. XG, YX, and YY collected, analyzed and interpreted the experimental data. JL, TL, and XZ revised the manuscript for important intellectual content. All authors contributed to the article and approved the submitted version.

## Funding

This work was supported by Wu Jieping Medical Foundation (320.6750.2020-17-7).

## Conflict of Interest

The authors declare that the research was conducted in the absence of any commercial or financial relationships that could be construed as a potential conflict of interest.

## Publisher’s Note

All claims expressed in this article are solely those of the authors and do not necessarily represent those of their affiliated organizations, or those of the publisher, the editors and the reviewers. Any product that may be evaluated in this article, or claim that may be made by its manufacturer, is not guaranteed or endorsed by the publisher.

## Supplementary Material

Supplementary Figure 1 Somatic mutation spectrum of five lesions in three patients. **(A, B)** Somatic SNV/INDEL mutation spectrum; **(C)** Somatic CNV mutation spectrum. C1T1A: II-2 tumor A; C1T2A: II-2 tumor B; C2T2A: II-4 tumor A; C2T2B: II-4 tumor B; C3T3: II-5.
