# Supplementary material for: A Germline Mutation in ATR Is Associated With Lung Adenocarcinoma in Asian Patients
Source: Front Oncol. 2022 May 31;12:855305. doi: 10.3389/fonc.2022.855305 (PMC9195140; doi:10.3389/fonc.2022.855305)
Supplement: Supplementary Figure 1 — Somatic mutation spectrum of five lesions in three patients. (A, B) Somatic SNV/INDEL mutation spectrum; (C) Somatic CNV mutation spectrum. C1T1A: II-2 tumor A; C1T2A: II-2 tumor B; C2T2A: II-4 tumor A; C2T2B: II-4 tumor B; C3T3: II-5. [file DataSheet_1.pdf]

Supplementary Figures

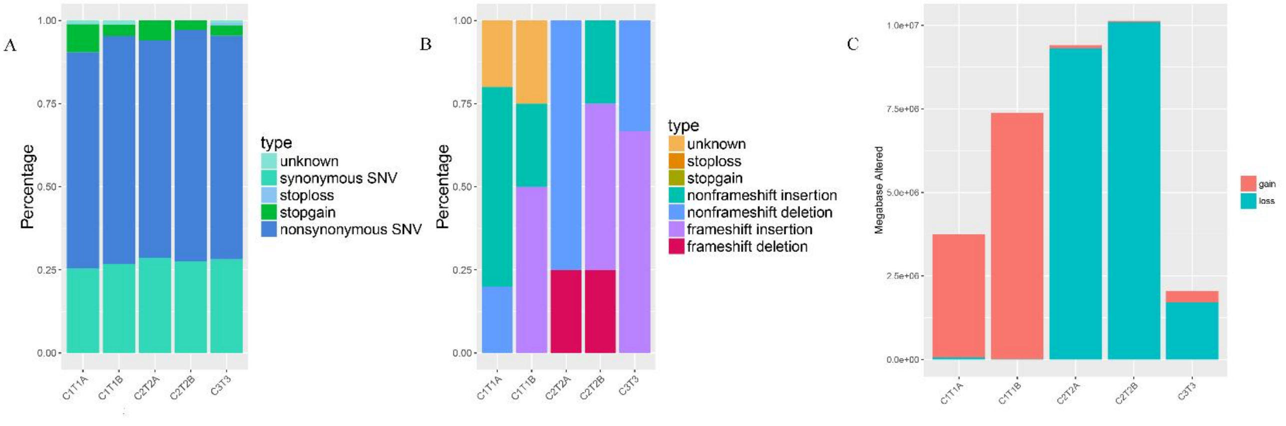

Figure S1. Somatic mutation spectrum of five lesions in three patients. (A-B) Somatic SNV/INDEL mutation spectrum; (C) Somatic CNV mutation spectrum. C1T1A: Ⅱ-2 tumor A; C1T2A: Ⅱ-2 tumor B; C2T2A: Ⅱ-4 tumor A; C2T2B: Ⅱ-4 tumor B; C3T3: Ⅱ-5
